# Efficiacy of injection specific immunotherapy for grass pollen seasonal allergic rhinitis

**DOI:** 10.1186/1939-4551-8-S1-A80

**Published:** 2015-04-08

**Authors:** Eliza Wasilewska, Joanna Orlicz-Widawska

**Affiliations:** 1 Medical University of Gdansk, Poland; 2 Private Practice, Poland

## Background

Allergy to pollen grass causes significant morbidity in Poland. Allergic rhinitis is a common condition which, at its most severe, can significantly impair quality of life despite treatment with antihistamines and topical nasal corticosteroids.

The aim of the study was to evaluate the efficiacy of injection specific immunotherapy (SIT) mixture grass pollen allergoide at 20 000 AUM/ml for seasonal allegic rhinitis.

## Methods

72 patients followed 12-month immunotherapy and 63 patients were observed without immunotherapy.Clinical efficacy was based on symptom and medication scores and the percentage of healthy days (days without symptoms or medication). Severity of rhinitis scales, visual analogue scale, evaluation of the treatment by doctors and patients, immediate and delayed cutaneous response and quality of life questionnaires were also studied.

## Results

The patients with immunotherapy showed decrease in symptoms (p = 0.01), medication (p = 0.003) and both (p =0.001), increase of healthy days (p=0.02) one year after treatment. Rhinitis severity scales decreased after immunotherapy. Both clinical evaluation by physicians and patient’s self-evaluation showed efficacy in 81% and 74% of the patients with SIT. None of these changes were observed in group without SIT. Immediate cutaneous response was significantly reduced in SIT patients compare to other patients, one year after SIT.

## Conclusions

A recombinant allergen vaccine can be a effective and safe treatment to reduced symptoms of allergic rhinitis.

